# Brief educational video plus telecare to enhance recovery for older emergency department patients with acute musculoskeletal pain: study protocol for the BETTER randomized controlled trial

**DOI:** 10.1186/s13063-020-04552-3

**Published:** 2020-07-06

**Authors:** Timothy F. Platts-Mills, Samuel A. McLean, Morris Weinberger, Sally C. Stearns, Montika Bush, Brittni B. Teresi, Karen Hurka-Richardson, Kurt Kroenke, Robert D. Kerns, Mark A. Weaver, Francis J. Keefe

**Affiliations:** 1 Department of Emergency Medicine, University of North Carolina at Chapel Hill, Houpt Bldg, 170 Manning Dr, Chapel Hill, NC 27599 USA; 2 Department of Anesthesiology, University of North Carolina Hospitals, Chapel Hill, NC USA; 3 Department of Health Policy and Management, Gillings School of Public Health, University of North Carolina at Chapel Hill, Chapel Hill, NC USA; 4 Regenstrief Institute and Department of Medicine, Indiana University, Indianapolis, IN USA; 5 School of Medicine, Yale University, New Haven, CT USA; 6 Department of Mathematics and Statistics, Elon University, Elon, NC USA; 7 Department of Psychology and Neuroscience, Duke University, Durham, NC USA

**Keywords:** Musculoskeletal pain, Geriatrics, Emergency medicine, Shared decision making

## Abstract

**Background:**

Chronic musculoskeletal pain (MSP) affects more than 40% of adults aged 50 years and older and is the leading cause of disability in the USA. Older adults with chronic MSP are at risk for analgesic-related side effects, long-term opioid use, and functional decline. Recognizing the burden of chronic MSP, reducing the transition from acute to chronic pain is a public health priority. In this paper, we report the protocol for the Brief EducaTional Tool to Enhance Recovery (BETTER) trial. This trial compares two versions of an intervention to usual care for preventing the transition from acute to chronic MSP among older adults in the emergency department (ED).

**Methods:**

Three hundred sixty patients from the ED will be randomized to one of three arms*: full intervention* (an interactive educational video about pain medications and recovery-promoting behaviors, a telecare phone call from a nurse 48 to 72 h after discharge from the ED, and an electronic communication containing clinical information to the patient’s primary care provider); *video-only intervention* (the interactive educational video but no telecare or primary care provider communication); or *usual care*. Data collection will occur at baseline and at 1 week and 1, 3, 6, and 12 months after study enrollment. The primary outcome is a composite measure of pain severity and interference. Secondary outcomes include physical function, overall health, opioid use, healthcare utilization, and an assessment of the economic value of the intervention.

**Discussion:**

This trial is the first patient-facing ED-based intervention aimed at helping older adults to better manage their MSP and reduce their risk of developing chronic pain. If effective, future studies will examine the effectiveness of implementation strategies.

**Trial registration:**

ClinicalTrials.gov NCT04118595. Registered on 8 October 2019.

## Administrative information

**Table:** 

Title	Brief Educational Video plus Telecare to Enhance Recovery for Older Emergency Department Patients with Acute Musculoskeletal Pain: Study Protocol for the BETTER Randomized Controlled Trial
Trial registration	ClinicalTrials.gov, NCT04118595, Registered October 8, 2019
Protocol version	Issue date: 23 Oct 2019 Protocol amendment number: 01
Funding	National Institute on Aging of the National Institutes of Health under award number 1R01AG058702-01A1
Author details	Timothy F. Platts-Mills, MD, MSc^1^, Samuel A. McLean, MD, MPH^2^, Morris Weinberger, PhD^3^, Sally C. Stearns, PhD^3^, Montika Bush, PhD^1^, Brittni B. Teresi, BA^1^, Karen Hurka-Richardson, NP^1^, Kurt Kroenke, MD, MACP^4^, Robert D. Kerns, PhD^5^, Mark A. Weaver, PhD^6^, Francis J. Keefe, PhD^7^ ^1^ Department of Emergency Medicine, University of North Carolina at Chapel Hill, Chapel Hill, NC, USA. ^2^Department of Anesthesiology, University of North Carolina Hospitals, Chapel Hill, NC, USA. ^3^Department of Health Policy and Management, Gillings School of Public Health, University of North Carolina at Chapel Hill, Chapel Hill, NC, USA. ^4^ Regenstrief Institute and Department of Medicine, Indiana University, Indianapolis, IN, USA. ^5^School of Medicine, Yale University, New Haven, CT, USA. ^6^Department of Mathematics and Statistics, Elon University, Elon, NC, USA. 7Department of Psychology and Neuroscience, Duke University, Durham, NC, USA.
Name and contact information for the trial sponsor	Tim Platts-Mills MD, MSc University of North Carolina-Chapel Hill Houpt Bldg, 170 Manning Dr, Chapel Hill, NC 27599 Phone: 559-240-6073 Email: tim_platts-mills@med.unc.edu
Role of sponsor	University of North Carolina-Chapel Hill is responsible for study design; collection, management, analysis and interpretation of the data; writing of the report; and the decision to submit the report for publication. Duke University, Elon University, Indiana University, and Yale University are serving as collaborators and advisors the study activities.

## Introduction

### Background and rationale

Musculoskeletal pain (MSP) is estimated to affect more than 40% of US adults age 50 years and older and results in more than $200 billion in healthcare costs annually [1, 2]. Chronic MSP is typically defined as pain affecting the bones, muscles, ligaments, tendons, or nerves for 3 months or more [3, 4]. Older adults are at particularly high risk for chronic MSP. Chronic MSP results in a greater reduction in physical function in older than younger adults and increases risk of falls and injury [5–7]. Chronic MSP also increases the risk of long-term opioid use, opioid use disorder, and overdose [8, 9].

Chronic MSP generally begins with an acute pain episode that persists. For example, approximately 9% of individuals transition from acute to chronic MSP following an episode of acute low back pain, [10] ~ 26% after a motor vehicle collision [11], and 5% or more after surgery [12]. Because of the debilitating consequences of chronic MSP, the National Institutes of Health (NIH) has identified developing better methods of preventing the transition from acute to chronic MSP as a public health priority [13, 14].

Several sources of evidence suggest that acute MSP interventions can reduce the transition to chronic MSP [15, 16]. In a quasi-experimental study of older adults receiving orthopedic surgery, optimizing analgesia after surgery reduced pain and improved function at 6 months [17]. Effective early treatment of acute MSP may prevent neurobiological changes mediating the development of chronic MSP [18, 19]. Observational studies of older adults suggest that acute MSP symptoms resolve in most individuals in the first 6 weeks, after which recovery is much less likely [16, 20]. For older adults who present for care with moderate or severe acute MSP, primary care follow-up often does not occur for several weeks [21, 22]. Thus, the initial presentation for acute MSP constitutes a unique opportunity to maximize recovery during a critical period of transition to recovery vs. pain persistence.

### Objectives and trial design

**Fig. 1:**
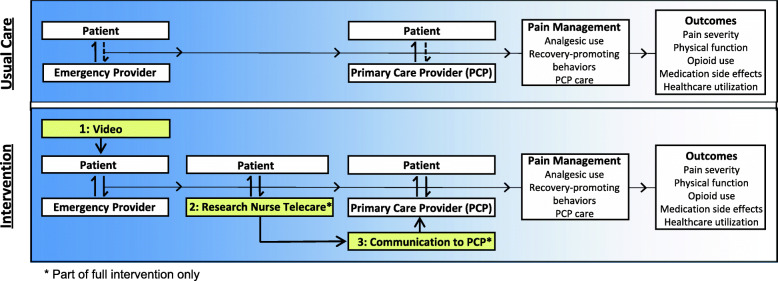
Conceptual model of shared decision-making during usual care and during the intervention for the proposed study. Bidirectional arrows represent a decision-making interaction between the patient and provider. Broken arrows between patients and providers indicate communication by patients who have not received education on pain management approaches

We have developed an ED-based intervention for patients with acute MSP based on a shared decision-making (SDM) model [23]. This intervention supports SDM between providers and patients at 3 or more points during the early recovery period (Fig. 1). The full intervention provides patients with information about pain management, obtains information from the patient about pain symptoms and early management choices, and supports discussion between patients and providers regarding the best course of treatment (bidirectional arrows, Fig. 1). SDM is an appropriate model for the management of acute MSP because there are numerous reasonable treatment options and the best option often depends on a patient’s values and preferences. Additionally, observational studies suggest that SDM during the ED encounter improves pain recovery and increases satisfaction with treatment [24, 25]. In this paper, we describe the methods of a patient-level, three-arm randomized controlled trial in which we compare two versions of an intervention, the full intervention (video, telecare, and PCP communication) and a video-only version, to usual care to prevent the transition to chronic MSP among older adults who present with MSP to an acute care setting.

## Methods: participants, interventions, and outcomes

### Overview of study design

The BETTER trial is an NIH-funded, assessor-blinded, randomized controlled trial approved by the Institutional Review Board at the University of North Carolina-Chapel Hill. ED patients aged 50 years and older presenting to the ED with acute MSP are screened for eligibility. All eligible and willing patients sign a consent form given to them by an RA in the ED, which includes permission to communicate with their PCP, as well as a Health Information Privacy form, which authorizes the research team to access their medical record.

**Fig. 2:**
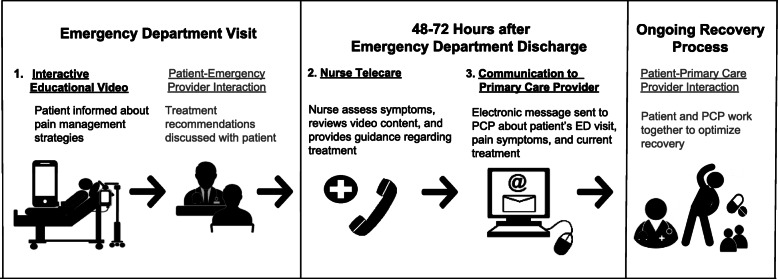
Description of the three components of the full intervention for the BETTER trial. Light gray text describes additional interactions between patients and provider that may be indirectly influenced by the intervention

Patients are randomized to one of three arms (Table 1). Those receiving the full intervention view an interactive educational video and receive a telecare phone call from a research nurse 48–72 h after study enrollment; in addition, communication about the patient’s ED visit and the treatment plan will be sent to their PCP electronically (Fig. 2). A second group of patients receive only the educational video, and a third group (the control arm) receive care as usual.

**Table 1:** Schedule of enrollment and interventions

	Enrolment	Allocation	In ED	48–72 h after discharge	
Time point	***-t***_***1***_	0	***Video***	***Telecare***	***PCP Communication***
**Enrolment**					
**Eligibility screen**	X				
**Informed consent**	X				
***Baseline interview***	X				
**Allocation**		X			
**Interventions**					
***Full intervention***			X	X	X
***Video-only***			X		
***Usual care***					

See Table 3 for assessments/data collection measures by study time point

All patients complete a baseline research assessment in the ED, a phone call at 1 week post-discharge to assess processes that might mediate recovery, and phone calls at 1, 3, 6, and 12 months following study enrollment to assess outcomes. The primary outcome is pain intensity and interference. Secondary outcomes include measures of physical function, opioid use, analgesic side effects, overall health, and healthcare utilization.

### Study setting and eligibility criteria

The study enrolls 360 patients aged 50 years and older who present to an acute care setting (ED or urgent care) in the University of North Carolina Hospitals healthcare system in the USA with MSP. Patients are excluded if they do not speak English, have been admitted to the hospital in the past 30 days, have pain due to self-injury, have an Emergency Severity Index score of one, are a prisoner, or have a diagnosis of somatoform disorder, schizophrenia, dementia, or bipolar disorder. Patients are also excluded if their pain is not musculoskeletal, such as pain due to ischemia or infection, or if their pain is located in the head, chest, or abdomen.

Patients who meet initial eligibility requirements are approached by the Research Assistant (RA) for an in-person assessment of additional exclusion criteria: current pain intensity rating < 4 (numeric pain rating scale: 0 = no pain, 10 = worst pain imaginable) or for those who have received pain medication, a pain intensity rating < 4 prior to receipt of pain medication, pain that began > 7 days prior to the ED visit, previous medical care for their pain, opioid use in the past 3 months (excluding the day of the ED visit), residence in a nursing home or homelessness, or at-risk alcohol use (≥ 5 drinks in a single day or ≥ 15 drinks in a single week). Patients must also have a working phone number for follow-up calls. Patients meeting all eligibility are invited to enroll in the study.

### Assignment of interventions: allocation and blinding

Patient randomization utilizes 1:1:1 allocation with randomly permuted blocks of random sizes. Randomization is stratified based on two dichotomous characteristics: age (50–64 years or ≥ 65 years) and access to a PCP (yes or no). The latter is assessed with the question, “Do you have access to a primary care provider who you can arrange a follow-up appointment within the next two weeks?” The study statistician created a program for generating the randomization schedule in SAS, version 9.4 (SAS Institute, Cary, NC). The Lead Data Manager updated the random number generation seed in the Statistician’s program, generated the final randomization schedule, and uploaded the resulting schedule into the secure randomization module in the Research Electronic Data Capture (REDCap) database. To ensure allocation concealment, the Lead Data Manager is the only member of the study team with access to the final randomization schedule. Throughout data collection, the primary RA collecting all follow-up data and the study statistician are blinded to patient treatment arm.

After baseline data is collected, a second unblinded RA randomizes the patient and meets with the patient to either show them the educational video or to conclude the acute care portion of the study. There is the potential for unblinding during follow-up interviews if a patient refers to intervention components. The primary outcome is assessed at the beginning of each interview to reduce the likelihood of unblinding for this outcome. If unblinding occurs, a different RA will complete that patient’s subsequent follow-up calls.

### Interventions

#### Full intervention

The full intervention includes three components.

##### Interactive educational video

The video was created in partnership with a local video production company (Horizon Productions, Durham, NC). The goal of the video is to educate patients and provide them with options for managing pain on their own. The video first discusses when and how to use the most common classes of pain medications (acetaminophen, nonsteroidal anti-inflammatory drugs (NSAIDs), and opioids) and then discusses recovery-promoting behaviors (physical activity, sleep, social support, and relaxation breathing; Table 2). To encourage engagement with the video, after each section, patients are prompted by the video to answer a multiple-choice question about the content and provided with an explanation of the correct response. The average time to show all sections of the video is 13 min and 20 s. The video is shown to patients after the initial evaluation by a provider but prior to discharge. The intent is that having seen the video, patients will be more likely to engage in shared decision-making with their provider regarding outpatient pain management prior to discharge.

**Table 2:** Outline of content provided in the educational video

Topic	Content
Introduction	Benefits and risks of treatment
Pharmacologic treatment	
Acetaminophen	Contraindications, risks, maximum dosage, names, knowledge question
NSAIDs	Contraindications, risks, common names, knowledge question
Opioids	Risks, side effects, side effect prevention, addiction, knowledge question
Strategies for using analgesics	Medication interactions; round the clock vs. as-needed; alternative therapies; consider pain medication before physical activity
Non-pharmacological treatment	
Physical activity	Movement to promote healing; physical therapy
Sleep	Prioritization; methods to improve sleep hygiene
Social support	Inform others about pain; seek support to stay active
Relaxation strategies	When and how to perform deep breathing exercises
Closing	Assess pain daily and modify approach as needed; encourage primary provider follow-up

Patients are shown the video on an iPad and provided with headphones. An unblinded RA watches the video with the patient and records the video play-through rate. When interrupted, the RA stops the video for the provision of healthcare and then continues when care is completed. If people (i.e., family or caregivers) are with the patient, the RA asks the patient if they would like these individuals to remain present before showing the video and documents the number of individuals who decide to remain in the room.

##### Telecare phone call from the research nurse

The telecare component is adapted from previous telecare trials aimed at optimizing pharmacological and behavioral treatment of pain in primary care and specialty settings [41–43]. In the BETTER trial, this component consists of a ~ 15-min phone call by a trained research nurse 48 to 72 h after the patient’s ED discharge. The goal of the call is to address suboptimal pain management treatments and behaviors and provide patients with knowledge to self-manage their pain symptoms. In designing the language for the call, we had four priorities: (1) reinforcing the video’s content, (2) identifying and addressing potentially unsafe analgesic use, (3) identifying and addressing barriers to using recovery promoting behaviors, and (4) ensuring PCP follow-up, particularly for those with persistent pain symptoms who may be at high-risk for a transition from acute to chronic pain.

The telecare conversation follows a standardized script that assesses pain symptoms; medication use, dosing, contraindications, and side effects; and use of and barriers to use of recovery promoting behaviors (physical activity, sleep, social support, and relaxation breathing). The language and content of the telecare call is designed to support a partnership between the patient and the nurse with an emphasis on respect for the patient’s experience and perspective, an explicit desire to understand what is important to the patient, and recognition that the patient will be making most treatment decisions without direct input from a medical provider. Consistent with this approach, the script utilizes open-ended questions, such as “what are you doing to manage your pain and get back to your usual activities?” rather than yes or no questions. In order to optimize the therapeutic value of the call, training for calls emphasizes supporting decisions and behaviors already undertaken by the patient that promote recovery. When a barrier, challenge, or problem is reported, the nurse acknowledges the problem and then direct the conversation to supporting the patient in identifying solutions. If indicated, the nurse may recommend use and dosing of acetaminophen, NSAIDs, and opioids. Since the nurse cannot prescribe opioids, nurse recommendations for use of opioids are limited to patients who received a prescription for opioids from the emergency provider. The call concludes with the research nurse encouraging PCP follow-up and providing information about how to obtain a PCP if the patient does not have one.

Several steps are taken to ensure adherence to the script. First, the nurse conducting the telecare call receives training from the investigative team on how to implement the script and completes three practice calls with feedback from the study principal investigator. Further, all telecare calls with patients are recorded and the duration of each call is tracked. The principal investigator for the study will listen to the first 25 calls and a random 10% sample of subsequent calls to assess adherence. If deviation from the duration, content, or style is observed, further education for the nurse is performed.

##### Communication with PCP

The final component is electronic communication with the patient’s PCP regarding the ED visit and treatment plan. The communication with the PCP is intended to support a transition of care from the ED provider to the PCP, allowing the PCP to reinforce the safe and effective use of analgesics and recovery-promoting behaviors and to adjust the treatment plan as needed. The communication with the PCP reflects our understanding of optimal pain management as an iterative process [44].

After completing telecare, the research nurse sends the patient’s PCP a summary of their condition and treatment plan, reporting date, time, location, and reason for the ED visit; results of ED diagnostics studies; discharge prescriptions and recommendations; a summary of the video, including the link; a summary of the telecare conversation including pain symptoms, medication use, and use of recovery promoting behaviors; and encouragement for follow-up and engagement with the patient regarding pain symptoms, safe and effective use of medication, and reinforcement of the need for recovery promoting behaviors. Messages are sent through an electronic message in the electronic health record to providers who use Epic, a common electronic health record system. For providers who do not use Epic, messages are sent through secure email or fax.

#### Video-only intervention

Patients in this arm view the interactive educational video as described above; however, they do not receive a telecare call from the research nurse nor is a message sent to their PCP. The reason for testing a video-only version of the intervention is that it is easier to implement, so if the video-only version has greater efficacy compared to usual care, then the video-only version may be the preferred approach for implementation efforts. Alternatively, a hybrid approach that uses either the full intervention or the video-only intervention based on patient factors that predict response to treatment might be developed.

#### Usual care

Patients randomized to the usual care arm receive analgesic recommendations, prescriptions, and advice regarding recovery behaviors as provided by the patient’s acute care provider and nurse. We recognize that the amount of time spent by providers and nurses on education about pain management during an acute care visit is varied but often brief. We have chosen not to include a sham intervention or enhanced usual care because the intent of the study is to determine if the intervention improves upon what is typically done for patients [45].

#### Provisions for clinical care

Consistent with this intent, no restrictions are placed on the care from acute care providers for any of the study arms. Discontinuation of interventions is not anticipated since this is an education-based intervention. However, participants may request to stop the study at any time. If a medication or behavior recommended in the video or telecare causes a concerning side effect for the participant, the participant will be advised to stop the behavior or medication and consult their PCP. Patients are compensated for their participation throughout the study, and there is no post-trial care upon completion of the study.

### Data collection and management

Patients complete a baseline interview in the ED to assess their (1) history of pain symptoms and current pain, (2) attempted treatments to manage pain including both analgesics and recovery-promoting behaviors, (3) perceived efficacy in their ability to manage pain, and (4) overall health (Table 3). Efficacy outcomes are assessed by phone 1, 3, 6, and 12 months after study enrollment (Table 3). Patients who are unexpectedly admitted to the hospital have their first follow-up call 1 week after hospital discharge. All 360 enrolled patients are compensated for their time for completing the assessments, receiving $40 for completing the ED assessment, $20 for the 1-week follow-up, and $25 for each additional follow-up.

**Table 3:** Data collection measures by study time point

Measure	Baseline (ED visit)	1-week follow up	1-, 3-, 6-, and 12-month follow-up
Access to a primary care provider	X		
Analgesic use (opioid and non-opioid)	X		
Use of recovery-promoting behaviors	X	X	X
Brief Pain Inventory (pain severity only) [26]	X		
Brief Pain Inventory (pain severity and interference)		X	X
Anticipated pain recovery [26]	X		
History of chronic pain [27]	X		
Pain Catastrophizing Scale^5^	X		
PROMIS Global Health-2a (prior to pain) [28]	X		
PROMIS Physical Function (prior to pain)	X		
PROMIS Global Health-2a (including pain)	X		X
PROMIS Physical Function (including pain)	X		X
Patient Health Questionnaire (prior to pain) [29]	X		
Generalized Anxiety Disorder-2 (prior to pain) [30]	X		
ENRICHD Social Support Instrument-2 questions [31]	X		
Control Preference Scale [32]	X		
Pain Self-Efficacy Questionnaire-4 item [33]	X	X	
Single Item Literary Screen [34]	X		
Tobacco Screening Measure [35]	X		
Global Impressions of Change [36]		X	X
9-item Shared Decision-making Questionnaire [37]		X	
Preparedness and confidence questions		X	
Opioid specific questions^a^		X	
Non-opioid analgesic questions		X	X
Opioid-Related Symptom Distress Scale [38]		X	X
Pittsburgh Insomnia Rating Scale [39]		X	
International Physical Activity Questionnaire [40]		X	
Health utilization questions^a^		X	X

^a^Data obtained through Electronic Health Record AND Questionnaires

^b^Data will be collected for all intervention groups

#### Data quality and safety monitoring

Data is entered into REDCap, a secure research database which assigns patients unique identifiers to maintain confidentiality, by an RA. Any paper forms, including signed consent and HIPPA forms and data collection forms, will be maintained by the Study Coordinator in a locked filing cabinet. The Lead Data Manager runs weekly reports to assess data quality. As the trial is low risk and does not require a Data Safety Committee, the Data Safety Officer, who is independent from the study team, will track participant recruitment and enrollment milestones and assess adverse events. The Data Safety Officer will receive both bi-annual reports and will be notified any time a serious adverse event occurs. If concerns arise, the Data Safety Officer, Study Coordinator, and PI will meet to discuss protocol modifications and communicate them to co-investigators and participants as needed.

#### Assessment and collection of outcomes

The primary outcome of pain severity and pain interference is assessed using the 11-item Brief Pain Inventory (BPI) at 1, 3, and 6 months. Secondary outcomes include physical function (PROMIS-Physical Function 4), analgesic use and side effects (Opioid-Related Symptom Distress Scale), overall health (PROMIS Global Health-Physical 2a), and individual components of the BPI (Table 2). Data on the use of recovery-promoting behaviors are collected through asking patients about strategies used besides medication in the last 2 weeks to manage pain. Anxiety symptoms will be measured using the Generalized Anxiety Disorder-Short Form and depressive symptoms using the Patient Health Questionnaire. The same data are collected at 1, 3, 6, and 12 months. Recognizing the potential for loss to follow-up and unrelated health events to influence outcomes at 12 months, data from this time point are not used for the primary outcome but are included in secondary analyses.

Healthcare utilization is measured by each patient’s total number of ED visits, total number of outpatient physician visits (including visits to an urgent care, primary care, or specialist), and total number of hospitalizations and days hospitalized. Data on healthcare utilization are collected using two approaches: (1) patient report and (2) review of electronic health record (EHR) data. Recognizing that we have incomplete access to EHRs for patients whose primary care is not within our healthcare system, patient reported healthcare utilization will be the primary source of information for the economic analysis. If there are sufficient patients with EHR data, then a separate subgroup analysis will be conducted in these patients.

### Statistical methods

We calculated power for comparing mean change in the BPI total score across the three trial arms using a standard approach for linear mixed models [46]. Following a recent consensus group recommendation, a 1 point difference in BPI is considered the minimum clinically important difference between treatment groups [47]. We simulated a study dataset in which the full intervention and video-only groups experienced post-randomization mean BPI scores 1 point lower than that in the usual care group. In the simulated data, we assumed that loss-to-follow-up times would be exponentially distributed such that the total loss would be 10% at 6 months. Under these assumptions, randomizing 120 patients to each group would provide at least 90% power for testing the overall null hypothesis of no difference across the three groups in mean change from baseline to any follow-up visit. Furthermore, this sample size would provide about 88% power for the comparison between the intervention group and the usual care group under these same assumptions, each tested at Bonferroni-adjusted 2.5% significance levels.

Linear mixed models will be used in the primary analysis with intention-to-treat group assignments, controlling for baseline variables that are known to influence pain recovery: ED pain, age, gender, race, comorbidities, baseline intermittent opioid use, history of chronic pain, and access to a PCP. We will first test a 6 degrees of freedom (df) linear contrast of the overall null hypothesis of no mean difference in the primary outcome at any of the 1-, 3-, and 6-month follow-up assessments across the 3 groups at the 5% significance level. If the overall null hypothesis is rejected, we will then conduct separate 3 df contrasts to compare each of the intervention groups with the control group using a Bonferroni-adjusted 2.5% significance level. We will secondarily estimate mean pairwise differences along with 95% confidence intervals at each time point, including data collected at the 12-month follow-up time point. We will conduct secondary analyses to estimate the effect of treatment on secondary outcomes (physical function, opioid use, side effects, sleep quality, anxiety symptoms, depressive symptoms, overall health, and healthcare utilization) using a similar approach. A per-protocol analysis, which will exclude patients who were found to meet exclusionary criteria after enrolling in the study, specifically those who are admitted to the hospital and who are found to have pain that is not musculoskeletal in origin, will also be performed for all outcomes.

**Fig. 3:**
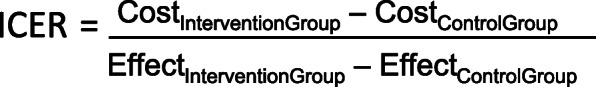
Incremental cost-effectiveness ratio

To estimate the change in healthcare cost resulting per case of moderate or severe pain prevented with the full or the video-only intervention, we will calculate an incremental cost-effectiveness ratio (Fig. 3) [48–50]. Bootstrapping will be used to address uncertainty [51, 52]. Costs in the numerator are estimated from key patient-reported health utilization costs (physician visits, ED visits, and days hospitalized) for both intervention and usual care groups. Intervention costs for the numerator will be the per patient costs of showing the video and administering the telecare call, which includes estimates for compensating nurses for their time. The denominator will be the likelihood of preventing a case of moderate or severe pain. If a substantial portion of patients appear to receive all their care from sites where data are available in the EHR, additional cost analyses in this subset of patients will be conducted using only EHR data.

## Discussion

The BETTER trial is a three-arm efficacy trial of two versions of an SDM-based intervention for older adults receiving care for acute MSP. Our goal is to evaluate the effects of the full intervention and video-only intervention on long-term pain severity and pain interference compared to usual care; secondarily, it assesses the effect on analgesic-related side effects and adverse events, function, quality of life, long-term opioid use, and healthcare utilization. Although the full intervention includes multiple components of SDM (patient education, discussion between nurse and patient, communication with primary provider to support additional care), the educational video on its own would be more feasible for implementation and is therefore included as one of the trial arms. In the pilot study for BETTER, clinically important differences in pain symptoms and opioid use between both versions of the intervention and the usual care group were observed at 1 month [53]. We will examine whether these differences are confirmed in this appropriately powered trial and whether the effects of the intervention are sustained beyond 1 month. Although older adults are the focus of this study, if efficacious, we intend to test the generalizability of the intervention(s) on other populations in other clinical settings.

The video we developed provides patients with information regarding medication and behaviors to inform their pain management decisions during conversations with their provider and after the visit. The video also encourages specific medications and behaviors which are likely to be beneficial during recovery. Understanding of analgesics and recovery-promoting behaviors among older adults is often poor, and patients who lack a basic understanding of analgesic names, dosing, and contraindications are at increased risk for side effects and adverse events [54]. Additionally, less formal education is associated with higher rates of opioid use [55, 56], suggesting that education regarding risks of opioids and of alternate pain management approaches might reduce opioid use. Fortunately, observational data suggest that patients want more communication with acute care providers about how to treat their pain [57]. A central assumption underlying the development of the intervention video is that increasing patient knowledge about analgesics and recovery behaviors improves recovery and reduces adverse events. Regarding opioid use, the intervention video teaches patients about the risks and benefits of opioids. It warns that opioids have inherent risks and should be avoided in patients at high risk for opioid use disorder, but that judicious use of opioids during the first few days of recovery may improve long-term outcomes for patients with acute severe pain [17, 58]. While analgesics can help reduce pain symptoms, exclusively focusing on analgesics as the means for optimizing recovery is neither the most effective approach nor a healthy message for patients. Sleep, social support, and management of symptoms of depression and anxiety are also likely important during the early recovery period [59–61]. Further, to the extent possible, physical activity during the early recovery period likely improves recovery [62, 63]. The telecare component of the full intervention is designed to help patients incorporate both proper analgesic use and recovery-promoting behaviors into pain management; it is informed by the work of Kroenke and colleagues, who have observed decreases in pain as a result of symptom monitoring and telecare management of symptoms for patients with chronic pain in both primary care and oncology settings [41–43].

Although we consider the study to be an efficacy trial, we made several deliberate choices to increase the clinical relevance of the study in order to facilitate subsequent implementation. First, the inclusion criteria broadly include patients with acute MSP and we anticipate the study sample will be diverse in regard to etiology of pain symptoms as well as race, ethnicity, and formal education. Second, the video and telecare are brief (approximately 15 min in duration) so that they could be administered during an acute care visit and by a nurse, respectively. Third, we decided to allow family members and friends to remain in the room during the viewing of the video, recognizing that this is what is likely to happen during implementation.

There are several anticipated challenges to the conduct of this trial. One challenge is loss to follow-up. In our prior work, follow-up rates ranged from 76 to 93% [11, 53]. Several strategies have been taken to optimize follow-up: we are testing phone numbers of patients prior to enrollment; collecting alternate phone numbers and alternate contacts; reminding all patients about follow-up calls by mail; and compensating patients for each call they complete. A second challenge is that chronic MSP can cause physical disability, sleep disruption, and social isolation [64, 65]. Simply teaching patients how to address these problems does not ensure they will be resolved. Therefore, the nurse is individualizing telecare as much as possible to support patients in identifying solutions. Similarly, the video provides options for recovery-promoting behaviors and encourages PCP follow-up when appropriate. Third, although the video is designed to promote an SDM-type conversation between providers and patients prior to discharge, whether patients use the knowledge they gain from the video to engage in conversations with providers and whether providers are receptive to these conversations are unknown. To measure the effect of the video on promoting SDM, patient-provider interactions are assessed during the 1-week process outcome assessment.

The NIH has declared a need to prevent the transition from acute to chronic MSP, especially among older adults. The BETTER trial is the first clinical trial to address this problem using an SDM approach in an acute care setting. Given the large number of older adults receiving care annually for acute MSP, the results of this trial have the potential to inform implementation and dissemination efforts that could have a positive impact on the burden of chronic MSP and long-term opioid use among older adults.

### Trial status

The trial is registered on ClinicalTrials.gov under NCT04118595. The study is using Study Protocol Version 1, October 23, 2019. The first participant was enrolled on February 3, 2020. The estimated completion date of recruitment is June 1, 2022.

## Supplementary information

**Additional file 1.** Informed consent

## Data Availability

Deidentified data will be shared on ClinicalTrials.gov 9 to 36 months following publication. Investigators who would like access to the full dataset may contact the PI directly.

## Abbreviations

BETTER: Brief Educational Tool to Enhance Recovery
BPI: Brief Pain Inventory
ED: Emergency department
EHR: Electronic health record
MSP: Musculoskeletal pain
NSAID: Nonsteroidal anti-inflammatory drug
PCP: Primary care provider
RA: Research Assistant
REDCap: Research Electronic Capture Database
SDM: Shared decision-making
