# Supplementary material for: Brief educational video plus telecare to enhance recovery for older emergency department patients with acute musculoskeletal pain: study protocol for the BETTER randomized controlled trial
Source: Trials. 2020 Jul 6;21:615. doi: 10.1186/s13063-020-04552-3 (PMC7336469; doi:10.1186/s13063-020-04552-3)
Supplement: Supplementary file 1 — Additional file 1. Informed consent [file 13063_2020_4552_MOESM1_ESM.docx]

**Appendix 1. Informed Consent**

**University of North Carolina at Chapel Hill Consent to Participate in a Research Study Adult Participants**

**Consent Form Version Date: 01/14/2019**

**IRB Study #** 19-0204

**Title of Study**: Reducing the Transition from Acute to Chronic Musculoskeletal Pain among Older Adults (BETTER from Pain)

**Principal Investigator**: Dr. Timothy Platts-Mills, MD

**Principal Investigator Department**: Emergency Medicine

**Principal Investigator Phone number**: (559) 240-6073

**Principal Investigator Email Address**[: tplattsm@med.unc.edu](mailto:tplattsm@med.unc.edu)

**Funding Source and/or Sponsor:** NIH National Institute on Aging (NIA)

**Study Contact Telephone Number**: (919) 445-6055

**Study Contact Email**: karen_hurkarichardson@med.unc.edu

Summary: The purpose of this study is to compare three different ways of helping people recover from musculoskeletal pain. Study participants will be randomly placed into one of three groups. The three groups will receive the following care: Group 1 – shown an educational video; Group 2 – shown an educational video and called by a nurse a few days after the ER visit; Group 3 – receive the care you would usually receive in the ER. We will compare the three groups based on pain scores. The goal is to understand the effect of the video either with or without the phone call on recovery. If you participate, your part in the study will last 1 year. We will call you to ask follow-up questions about your pain and health 1 week after your ER visit, then again at 1 month, 3 months, 6 months, and 12 months. This will help us see if the video or video and nurse phone call help patients over time.

**What are some general things you should know about research studies?**

You are being asked to take part in a research study. To join the study is voluntary.

You may choose not to participate, or you may withdraw your consent to be in the study, for any reason, without penalty.

Research studies are designed to obtain new knowledge. This new information may help people in the future. You may not receive any direct benefit from being in the research study. There also may be risks to being in research studies. Deciding not to be in the study or leaving the study before it is done will not affect your relationship with the researcher, your health care provider, or the University of North Carolina-Chapel Hill. If you are a patient with an illness, you do not have to be in the research study in order to receive health care.

Details about this study are discussed below. It is important that you understand this information so that you can make an informed choice about being in this research study.

You will be given a copy of this consent form. You should ask the researchers named above, or staff members who may assist them, any questions you have about this study at any time.

**What is the purpose of this study?**

The purpose of this research study is to compare three different ways of helping people recover from musculoskeletal pain. Musculoskeletal pain is common, and sometimes lasts for a long time. When it does, it’s hard on patients and can put them at risk of side effects from pain medications. The research team has developed an educational intervention with basic but important information about pain management. Study participants will be randomly placed into one of three groups. The three groups will receive the following care: Group 1 – shown an educational video; Group 2 – shown an educational video and called by a nurse a few days after the ER visit. During the phone call, you may receive advice from the nurse regarding behaviors and medications to manage your pain; Group 3 – receive the care you would usually receive in the ER. We will compare the three groups based on pain scores. The intent of the study is to understand whether the video or the video with the phone call can help patients manage their pain more effectively.

You are being asked to be in the study because you are age 50 and older and you have musculoskeletal pain.

**Are there any reasons you should not be in this study?**

You should not be in this study if you are pregnant, a prisoner, unable to understand English, do not have a phone, or have taken any opioids in the past 3 months.

**How many people will take part in this study?**

There will be approximately 400 people in this research study.

**How long will your part in this study last?**

Participation in the study will take a total of about 2 to 2.5 hours over the course of 1 year. The amount of time depends on which group you are in. After you consent, we will spend about 20 minutes asking you questions. If you are in the group of patients that is shown a video, viewing the video will take 15 minutes. Total time in the ER for these questions and video will be about 35 minutes. After that, if you are in the group that gets a nurse phone call, you will take part in a 15-minute phone conversation 2-3 days after the ER visit. The research nurse may give you advice about activities and medications that can help manage your pain. We will also call you around 1 week and 1, 3, 6, and 12 months after your ER visit. These calls will take about 20 minutes each.

**What will happen if you take part in the study?**

**During your visit to the ED:**

**Initial interview in the ER:**

After you consent, you will complete a 20-minute interview with me or another member of the study staff. This will involve answering questions about the pain you are experiencing and your health history.

**Group Assignment:**

After the initial interview, you will be assigned to one of three groups. You will receive your assignment randomly, like flipping a coin. One group of patients sees a 15-minute educational video and then receives a 15-minute call from a nurse 2-3 days after going home. The nurse will ask you about your pain and how you’ve been feeling since discharge. The nurse will also send your primary care provider a summary of your ER visit and phone call. The summary will include any recommendations the nurse has made for your pain treatment. The second group only sees the educational video. The third group will receive care as they normally would in the ER.

**Follow-up Phone Calls:**

After the ER visit, you will receive five telephone calls over the next twelve months. Someone from our study staff will call you to do these follow-up interviews. We will ask you questions about your pain and health. The phone calls will occur one week and 1, 3, 6 and 12 months after the date of your ER visit.

Each of these calls will take about 20 minutes. During these calls, you will talk about how you are feeling, your recovery, and any medications you are taking. You will get a reminder mailed to you before each of the calls. In the reminder letter we will also include a copy of the questions we will be asking you. This is to help you follow along during the phone interview. You don’t need to fill out the questionnaire, we will do that when we talk to you.

At any time during the study, you may choose to not answer a question for any reason. If you have questions at any point during the study, you may contact the study staff member listed on this consent form. You may also contact Dr. Tim Platts-Mills, who is the physician responsible for patients’ safety in this study.

**What are the possible benefits from being in this study?**

Research is designed to benefit society by gaining new knowledge. The benefits to you from being in this study may be that you learn new information about medical and non-medical pain management.

**What are the possible risks or discomforts involved from being in this study?**

All research involves some risk. A minimal risk in this study is psychological discomfort. You might feel uncomfortable talking about pain or side effects that you may be experiencing.

Researchers will be trained to minimize discomfort. There may be uncommon or previously unknown risks. You should report any problems to the research team.

A breach of confidentiality occurs if private information is unintentionally or intentionally shared with people outside of the study team. It is possible in any study in which personally identifying information is collected, like your name or birth date. In this study, patients will provide personal information regarding their health, pain status, medication use, and any hospital or primary care visits.

**If you choose not to be in the study, what other treatment options do you have?**

You do not have to be in this research study in order to receive treatment for your musculoskeletal pain at UNC. If you do not participate, you will receive the usual care for your condition.

**What if we learn about new findings or information during the study?**

You will be given any new information gained during the course of the study that might affect your willingness to continue your participation.

**How will information about you be protected?**

During screening, a research assistant conducting the interview will write responses on paper. Following the screening, the nurse will log the recorded data into a secure database and file the printed assessments in a secure location accessible only to research staff. The individual surveys will be labeled with a unique patient identifier. All paper copies of surveys will be kept in a locked file cabinet in the principal investigator’s office. All patient identifying information will be kept on a separate file on a password protected computer locked in the principal investigator’s office. Paper files will be shredded 3 years after study completion. Electronic data will be stored on REDCap until all analyses relevant to the study have been completed. One group of patients will receive a telephone follow-up call from a nurse 2-3 days after discharge from the hospital. Those telephone calls will be recorded. This will help researchers understand the kind of conversations patients have with nurses and to make sure that the protocol is followed as established. These conversations will be recorded and uploaded to a computer located in the PI’s office. There, they will be reviewed by the PI and study staff. Recordings will be stored in a password protected file with a unique patient identifier.

You may decide that you do not want your follow-up call to be recorded. There is no penalty if you decide you do not want your follow-up call to be recorded.

Following this telephone call, the nurse will enter a note into your electronic health record (EHR). They will use a standard template to update your primary care provider about your current status, the information you discussed, and any recommendations or referrals made.

This note will be sent to your primary care provider through the secure EHR.

If we cannot contact your PCP through the EHR, a secure email/fax will be sent to them.

Participants will not be identified in any report or publication about this study. We may use de- identified data from this study in future research without additional consent. Although every effort will be made to keep research records private, there may be times when federal or state law requires the disclosure of such records, including personal information. This is very unlikely, but if disclosure is ever required, UNC-Chapel Hill will take steps allowable by law to protect the privacy of personal information. In some cases, your information in this research study could be reviewed by representatives of the University, research sponsors, or government agencies (for example, the FDA) for purposes such as quality control or safety.

In some cases, your information in this research study could be reviewed by the UNC-Chapel Hill Institutional Review Board (IRB), the Office for Human Research Protections (OHRP) or the FDA for the purposes of quality control and the protection of human subjects.

**What will happen if you are injured by this research?**

There is minimal risk for injury by this research. However, all research involves a chance that something bad might happen to you.  This may include the risk of personal injury. In spite of all safety measures, you might develop a reaction or injury from being in this study. If such problems occur, the researchers will help you get medical care, but any costs for the medical care will be billed to you and/or your insurance company. The University of North Carolina at Chapel Hill has not set aside funds to pay you for any such reactions or injuries, or for the related medical care. You do not give up any of your legal rights by signing this form.

**What if you want to stop before your part in the study is complete?**

You can withdraw from this study at any time, without penalty. If you would like to immediately withdraw from the study, please notify any research staff that you would like to do so. Alternatively, you may contact Dr. Platts-Mills at tplattsm@med.unc.edu or (559) 240-6073 if you would like to request to be withdrawn from the study. If you provided information prior to withdrawing from the study, you may request that such information be destroyed.

The investigators also have the right to stop your participation at any time. This could be because you have had an unexpected reaction, or have failed to follow instructions, or because the entire study has been stopped.

**Will you receive anything for being in this study?**

You can receive up to $160 for taking part in this study.

Subject payments include $40 for completing initial ED evaluation, $20 for completing the 1- week follow-up, and $25 for completing follow-ups at 1 month, 3 months, 6 months, and 12 months. All payments will be made using reloadable Visa gift cards. The gift card will be loaded with $40 at the time of the ED visit. You will be given the card at the completion of the intervention. You will keep the card in your possession and the card will be re-loaded by research staff after the completion of each telephone call at 1 week, 1, 3, 6 and 12 months.

**Will it cost you anything to be in this study?**

It will not cost you anything to be in this study.

**Who is sponsoring this study?**

This research is funded by the National Institute of Health’s National Institute on Aging. This means that the research team is being paid by the sponsor for doing the study. The researchers do not, however, have a direct financial interest with the sponsor or in the final results of the study.

**What if you have questions about this study?**

You have the right to ask, and have answered, any questions you may have about this research. If you have questions about the study (including payments), complaints, concerns, or if a research- related injury occurs, you should contact the researchers listed on the first page of this form.

A description of this clinical trial will be available on [www.clinicaltrials.gov,](http://www.clinicaltrials.gov/) as required by U.S. Law. This website will not include information that can identify you. At most, the website will include a summary of the results. You can search this website at any time.

**What if you have questions about your rights as a research participant?**

All research on human volunteers is reviewed by a committee that works to protect your rights and welfare. If you have questions or concerns about your rights as a research subject, or if you would like to obtain information or offer input, you may contact the Institutional Review Board at 919-966-3113 or by email to [IRB_subjects@unc.edu.](mailto:IRB_subjects@unc.edu)

**Participant’s Agreement**:

I have read the information provided above. I have asked all the questions I have at this time. I voluntarily agree to participate in this research study.

| Signature of Research Participant | Date |
| --- | --- |
| Printed Name of Research Participant |  |

| Signature of Research Team Member Obtaining Consent | Date |
| --- | --- |
| Printed Name of Research Team Member Obtaining Consent |  |
